# Supplementary material for: Enterococcal Phages: Food and Health Applications
Source: Antibiotics (Basel). 2023 May 2;12(5):842. doi: 10.3390/antibiotics12050842 (PMC10215702; doi:10.3390/antibiotics12050842)
Supplement: Supplementary file 1 [file antibiotics-12-00842-s001.zip › antibiotics-2340538-supplementary.pdf]

Supplementary Table S1: Complete bacteriophage genomes used for the construction of the phylogenetic tree. The family, subfamily and genus if known are indicated. The origin, genome size in bp and accession number are also indicated. UC: Unclassified; NI: Not indicated.

**Name**	**Host**	**Family/ Subfamily**	**Genus**	**Origin**	**Genome Size**	**Accession**
ECP3	*E. faecalis*	*Herelleviridae/Brockvirinae*	*Kochikohdavirus*	NI	145518	KJ801817
EFLK1	*E. faecalis*	*Herelleviridae/Brockvirinae*	*Kochikohdavirus*	Sewage	130952	KR049063
MDA2	*E. faecium*	*Herelleviridae/Brockvirinae*	*Kochikohdavirus*	NI	140226	MW633168
PBEF129	*E. faecalis*	*Herelleviridae/Brockvirinae*	*Kochikohdavirus*	NI	144230	MN854830
phiEF24C	*E. faecalis*	*Herelleviridae/Brockvirinae*	*Kochikohdavirus*	NI	142072	AP009390
SW5	*E. faecalis*	*Herelleviridae/Brockvirinae*	*Kochikohdavirus*	Sewage	143759	ON286976
vB_EfaH_EF1TV	*E. faecalis*	*Herelleviridae/Brockvirinae*	*Kochikohdavirus*	Hospital wastewater	143507	MK268686
vB_EfaM_Ef2.1	*E. faecalis*	*Herelleviridae/Brockvirinae*	*Kochikohdavirus*	Sewage water	140938	MK693030
vB_EfaM_Ef2.3	*E. faecalis*	*Herelleviridae/Brockvirinae*	*Kochikohdavirus*	Sewage water	147289	MK721192
vB_EfaS_156	*E. faecalis*	*Herelleviridae/Brockvirinae*	*Kochikohdavirus*	Cheese	141133	ERZ1464534
vB_OCPT_Bob	*Enterococcus* spp.	*Herelleviridae/Brockvirinae*	*Kochikohdavirus*	Sewage	142921	ON113169
vB_OCPT_Car	*Enterococcus* spp.	*Herelleviridae/Brockvirinae*	*Kochikohdavirus*	Sewage	141483	ON113168
vB_OCPT_Carl	*Enterococcus* spp.	*Herelleviridae/Brockvirinae*	*Kochikohdavirus*	Sewage	143879	ON113167
EFDG1	*E. faecium/E. faecalis*	*Herelleviridae/Brockvirinae*	*Schiekvirus*	Sewage	147589	KP339049
EfsSzw-1	*E. faecalis*	*Herelleviridae/Brockvirinae*	Schiekvirus	Sewage water	150272	MH791397
GVEsP-1	*Enterococcus* spp.	*Herelleviridae/Brockvirinae*	*Schiekvirus*	River water	149913	MZ333462
PEf771	*E. faecalis*	*Herelleviridae/Brockvirinae*	Schiekvirus	Water	151052	MN241318
phiSHEF13	*E. faecium*	*Herelleviridae/Brockvirinae*	*Schiekvirus*	Wastewater	151389	OL799258
phiSHEF16	*E. faecium*	*Herelleviridae/Brockvirinae*	*Schiekvirus*	Wastewater	151935	OL799260
UTI-EfS3	*E. faecalis*	*Herelleviridae/Brockvirinae*	*Schiekvirus*	NI	150393	OL870611
vB_EfaH_163	*E. faecium/E. faecalis*	*Herelleviridae/Brockvirinae*	*Schiekvirus*	Human stool	150836	CAJDKA010000002
vB_OCPT_Ben	*E. faecalis*	*Herelleviridae/Brockvirinae*	Schiekvirus	Sewage	151985	MN027503
vB_OCPT_Bill	*Enterococcus* spp.	*Herelleviridae/Brockvirinae*	*Schiekvirus*	Sewage	147049	OM966901
vB_OCPT_Bop	*Enterococcus* spp.	*Herelleviridae/Brockvirinae*	*Schiekvirus*	Sewage	153454	ON125307
vB_OCPT_CCS1	*Enterococcus* spp.	*Herelleviridae/Brockvirinae*	*Schiekvirus*	Sewage	150888	ON113170
vB_EfaH_149	*E. faecalis*	*Herelleviridae/Brockvirinae*	UC	Cheese	142215	ERZ1464531
EF62phi	*E. faecalis*	*Podoviridae/UC*	UC	Human	30505	CP002495
SEsuP-1	*Enterococcus* spp.	*Podoviridae/UC*	UC	Faeces	39319	MZ333458
AE4_17	*E. faecalis*	*Rountreeviridae/Sarlesvirinae*	*Copernicusvirus*	Sewage	18477	NC_055866
MDA1	*E. faecium*	*Rountreeviridae/Sarlesvirinae*	*Copernicusvirus*	NI	18058	MW623430
idefix	*Enterococcus* spp.	*Rountreeviridae/Sarlesvirinae*	*Copernicusvirus*	NI	18168	NC_049937
N13	*E. faecalis*	*Rountreeviridae/Sarlesvirinae*	*Copernicusvirus*	NI	18449	ON352054
vB_EfaP_Ef6.2	*E. faecalis*	*Rountreeviridae/Sarlesvirinae*	*Copernicusvirus*	Sewage water	17966	MK721188
vB_EfaP_Ef6.3	*E. faecalis*	*Rountreeviridae/Sarlesvirinae*	*Copernicusvirus*	Sewage water	18136	MK721196

vB_EfaP_Ef7.2	*E. faecalis*	*Rountreeviridae/Sarlesvirinae*	*Copernicusvirus*	Sewage water	18737	MK721183
vB_EfaP_Ef7.3	*E. faecalis*	*Rountreeviridae/Sarlesvirinae*	*Copernicusvirus*	Sewage water	18818	MK721184
vB_EfaP_Ef7.4	*E. faecalis*	*Rountreeviridae/Sarlesvirinae*	*Copernicusvirus*	Sewage water	18415	MK721198
vB_EfaP_Efmus1	*E. faecalis*	*Rountreeviridae/Sarlesvirinae*	*Copernicusvirus*	Sewage water	17927	MK721195
vB_EfaP_Efmus2	*E. faecalis*	*Rountreeviridae/Sarlesvirinae*	*Copernicusvirus*	Sewage water	18366	MK721197
vB_EfaP_Efmus3	*E. faecalis*	*Rountreeviridae/Sarlesvirinae*	*Copernicusvirus*	Sewage water	18286	MK721185
vB_EfaP_Efmus4	*E. faecalis*	*Rountreeviridae/Sarlesvirinae*	*Copernicusvirus*	Sewage water	18186	MK721193
vB_EfaP_IME195	*E. faecalis*	*Rountreeviridae/Sarlesvirinae*	*Copernicusvirus*	Hospital sewage	18607	NC_028693
ZEF1	*E. faecalis*	*Rountreeviridae/Sarlesvirinae*	*Copernicusvirus*	Sewage	18454	MT747434
phiSHEF14	*E. faecium*	*Rountreeviridae/Sarlesvirinae*	*Minhovirus*	Wastewater	19390	OL799259
vB_EfaP_IME199	*E. faecium*	*Rountreeviridae/Sarlesvirinae*	*Minhovirus*	Hospital sewage	18838	NC_049931
vB_EfaP_Zip	*E. faecium/ E. faecalis*	*Rountreeviridae/Sarlesvirinae*	*Minhovirus*	Sewage	18742	NC_049930
vB_OCPT_Ump	*Enterococcus* spp.	*Rountreeviridae/Sarlesvirinae*	*Minhovirus*	Sewage	19707	ON113181
EFA-1	*E. faecalis*	*Siphoviridae/Autographiviridae*	*Studiervirinae*	NI	40712	MT350292
EFA-2	*E. faecalis*	*Siphoviridae/Autographiviridae*	*Studiervirinae*	NI	39964	MT350293
9183	*E. faecium*	*Siphoviridae/Andrewesvirinae*	*Denvervirus*	Wastewater	86301	NC_055907
nattely	*E. faecalis*	*Siphoviridae/Andrewesvirinae*	*Vipetofemvirus*	Wastewater	85669	MT119360
vipetofem	*E. faecalis*	*Siphoviridae/Andrewesvirinae*	*Vipetofemvirus*	Wastewater	85371	MT119361
VWF	*Enterococcus* spp.	*Siphoviridae/Andrewesvirinae*	*Vipetofemvirus*	NI	85865	LT546029
vB_EfaS_140	*E. faecalis*	*Siphoviridae/Andrewesvirinae*	UC	Cheese	85454	ERZ1462946
AUEF3	*E. faecalis*	*Siphoviridae*	*Efquatrovirus*	Sewage	41257	KJ127304
Ec-ZZ2	*E. faecium*	*Siphoviridae*	*Efquatrovirus*	Hospital sewage	41170	NC_031260
EfaCPT1	*E. faecalis*	*Siphoviridae*	*Efquatrovirus*	Sewage	40923	JX193904
EFap02	*E. faecalis*	*Siphoviridae*	*Efquatrovirus*	Sewage	39766	OL505084
heks	*E. faecalis*	*Siphoviridae*	*Efquatrovirus*	Wastewater	39708	MT119359
IME_EF3	*E. faecalis*	*Siphoviridae*	*Efquatrovirus*	Sewage	41687	KF728385
IME-EF4	*Enterococcus* spp.	*Siphoviridae*	*Efquatrovirus*	Sewage	40692	NC_023551
LY0322	*E. faecalis*	*Siphoviridae*	*Efquatrovirus*	NI	40934	MH193369
LY0323	*E. faecalis*	*Siphoviridae*	*Efquatrovirus*	NI	40876	MH375074
MSF2	*E. faecalis*	*Siphoviridae*	*Efquatrovirus*	NI	40880	MK982307
Nonaheksakonda	*E. faecalis*	*Siphoviridae*	*Efquatrovirus*	Wastewater	41994	MK125140
phiNASRA1	*E. faecalis*	*Siphoviridae*	*Efquatrovirus*	NI	40139	MG264739
phiSHEF10	*E. faecalis*	*Siphoviridae*	*Efquatrovirus*	Wastewater	41680	OL799256
phiSHEF11	*E. faecalis*	*Siphoviridae*	*Efquatrovirus*	Wastewater	40790	OL799257
phiSHEF2	*E. faecalis*	*Siphoviridae*	*Efquatrovirus*	Wastewater	41712	MF678788
phiSHEF4	*E. faecalis*	*Siphoviridae*	*Efquatrovirus*	Wastewater	41081	MF678789
phiSHEF5	*E. faecalis*	*Siphoviridae*	*Efquatrovirus*	Wastewater	41598	MF678790
PMBT2	*E. faecalis*	*Siphoviridae*	*Efquatrovirus*	Sewage water	41489	MG708276
SANTOR1	*E. faecalis*	*Siphoviridae*	*Efquatrovirus*	Sewage water	37933	KX284704
Sigurd	*E. faecalis*	*Siphoviridae*	*Efquatrovirus*	Wastewater	41811	MZ326865
vB_Efa29212_2e	*Enterococcus* spp.	*Siphoviridae*	*Efquatrovirus*	NI	41351	OP559177
vB_EfaS_271	*E. faecalis*	*Siphoviridae*	*Efquatrovirus*	Sewage	40197	MT520979
vB_EfaS_785CC	*E. faecalis*	*Siphoviridae*	*Efquatrovirus*	NI	40956	MZ272341
vB_EfaS_AL2	*E. faecalis*	*Siphoviridae*	*Efquatrovirus*	Wastewater	40836	MH203384

vB_EfaS_AL3	*E. faecalis*	*Siphoviridae*	*Efquatrovirus*	Wastewater	40789	MH203383
vB_EfaS_Ef5.1	*E. faecalis*	*Siphoviridae*	*Efquatrovirus*	Sewage	41141	MK721199
vB_EfaS_Ef5.2	*E. faecalis*	*Siphoviridae*	*Efquatrovirus*	Sewage	41418	MK721186
vB_EfaS_Ef5.3	*E. faecalis*	*Siphoviridae*	*Efquatrovirus*	Sewage	39115	MK721200
vB_EfaS_Ef5.4	*E. faecalis*	*Siphoviridae*	*Efquatrovirus*	Sewage	40685	MK721191
vB_EfaS_Ef6.1	*E. faecalis*	*Siphoviridae*	*Efquatrovirus*	Sewage	40429	MK721187
vB_EfaS_Ef6.4	*E. faecalis*	*Siphoviridae*	*Efquatrovirus*	Sewage	41133	MK721190
vB_EfaS_IME196	*E. faecalis*	*Siphoviridae*	*Efquatrovirus*	Hospital sewage	38886	NC_028990
vB_EfaS_LM99	*E. faecalis*	*Siphoviridae*	*Efquatrovirus*	Sewage	40203	MH355583
vB_EfaS_Max	*E. faecium/E. faecalis*	*Siphoviridae*	*Efquatrovirus*	Sewage	40975	MK360024
vB_EfaS_Paulomi	*E. faecalis*	*Siphoviridae*	*Efquatrovirus*	Sewage	41921	OL539449
vB_EfS_L1	*E. faecalis*	*Siphoviridae*	*Efquatrovirus*	NI	41151	OP254195
vB_OCPT_CCS4	*Enterococcus* spp.	*Siphoviridae*	*Efquatrovirus*	Sewage	40050	ON113176
VEsP-2	*Enterococcus* spp.	*Siphoviridae*	*Efquatrovirus*	River water	43177	MZ333461
ZXL	*E. faecalis*	*Siphoviridae*	*Efquatrovirus*	Sewage	40804	ON113334
phiFL1A	*E. faecalis*	*Siphoviridae*	*Phifelvirus*	NI	38764	NC_013646
phiFL1B	*E. faecalis*	*Siphoviridae*	*Phifelvirus*	NI	38989	GQ478082
phiFL1C	*E. faecalis*	*Siphoviridae*	*Phifelvirus*	NI	38721	GQ478083
phiFL2A	*E. faecalis*	*Siphoviridae*	*Phifelvirus*	NI	36270	GQ478084
phiFL2B	*E. faecalis*	*Siphoviridae*	*Phifelvirus*	NI	36826	GQ478085
phiFL3A	*E. faecalis*	*Siphoviridae*	*Phifelvirus*	NI	39576	NC_013648
phiFL3B	*E. faecalis*	*Siphoviridae*	*Phifelvirus*	NI	40275	GQ478087
47	*E. faecalis*	*Siphoviridae*	*Saphexavirus*	NI	57289	ON086985
BC-611	*E. faecalis*	*Siphoviridae*	*Saphexavirus*	NI	53996	AB712291
EFap05-1	*E. faecalis*	*Siphoviridae*	*Saphexavirus*	Sewage	56564	OL505085
EFKL	*E. faecalis*	*Siphoviridae*	*Saphexavirus*	Sewage	58343	OP831581
EF-P29	*E. faecalis*	*Siphoviridae*	*Saphexavirus*	Sewage	58984	KY303907
Entf1	*E. faecalis*	*Siphoviridae*	*Saphexavirus*	Sewage	58938	MK800154
IME_EF1	*E. faecalis*	*Siphoviridae*	*Saphexavirus*	Sewage	57081	KF192053
SAP6	*E. faecalis*	*Siphoviridae*	*Saphexavirus*	NI	58619	JF731128
SSsP-1	*Enterococcus* spp.	*Siphoviridae*	*Saphexavirus*	Faeces	57270	MZ333457
UTI-EfS7	*E. faecalis*	*Siphoviridae*	*Saphexavirus*	NI	56144	OL870612
vB_EfaS_EF1c55	*E. faecalis*	*Siphoviridae*	*Saphexavirus*	NI	55876	MN103542
vB_EfaS_Ef2.2	*E. faecalis*	*Siphoviridae*	*Saphexavirus*	Sewage	58400	MK721189
vB_EfaS_Ef7.1	*E. faecalis*	*Siphoviridae*	*Saphexavirus*	Sewage	58018	MK721194
vB_EfaS_HEf13	*E. faecalis*	*Siphoviridae*	*Saphexavirus*	NI	57811	MH618488
vB_EfaS_IME198	*E. faecalis*	*Siphoviridae*	*Saphexavirus*	Hospital sewage	58000	NC_029016
vB_EfaS_PHB08	*E. faecalis*	*Siphoviridae*	*Saphexavirus*	NI	55244	MK570225
vB_OCPT_CCS2	*Enterococcus* spp.	*Siphoviridae*	*Saphexavirus*	Sewage	57735	ON113173
vB_OCPT_PG11	*Enterococcus* spp.	*Siphoviridae*	*Saphexavirus*	Sewage	57092	ON113179
vB_OCPT_PG13	*Enterococcus* spp.	*Siphoviridae*	*Saphexavirus*	Sewage	57775	ON113180
vB_OCPT_PG2	*Enterococcus* spp.	*Siphoviridae*	*Saphexavirus*	Sewage	57485	ON113177
vB_OCPT_PG9	*Enterococcus* spp.	*Siphoviridae*	*Saphexavirus*	Sewage	57478	ON113178
vB_OCPT_SDS1	*Enterococcus* spp.	*Siphoviridae*	*Saphexavirus*	Sewage	55880	ON113171

vB_OCPT_SDS2	*Enterococcus* spp.	*Siphoviridae*	*Saphexavirus*	Sewage	57457	ON113172
vB_OCPT_Toy	*Enterococcus* spp.	*Siphoviridae*	*Saphexavirus*	Sewage	57304	ON113175
VD13	*E. faecalis*	*Siphoviridae*	*Saphexavirus*	NI	55113	KJ094032
9181	*E. faecium*	*Siphoviridae*	UC	Wastewater	71854	MT939240
9184	*E. faecium*	*Siphoviridae*	UC	Wastewater	44108	MT939242
EFAP-1	*E. faecalis*	*Siphoviridae*	UC	NI	21115	FJ792813
EFC-1	*E. faecalis*	*Siphoviridae*	UC	Sewage	40286	KJ608188
EFP1	*E. faecium*	*Siphoviridae*	UC	NI	37561	MN995824
EFRM31	*E. faecalis*	*Siphoviridae*	UC	NI	16945	GU815339
IME-EFm1	*E. faecium*	*Siphoviridae*	UC	Sewage	42597	NC_024356
IME-EFm5	*E. faecium*	*Siphoviridae*	UC	Sewage	42265	NC_028826
phiEf11	*E. faecalis*	*Siphoviridae*	UC	NI	42822	GQ452243
phiFL4A	*E. faecalis*	*Siphoviridae*	UC	NI	37856	NC_013644
Q69	*E. faecalis*	*Siphoviridae*	UC	Cheese	42141	ERZ1464538
vB_EfaS_159	*E. faecalis*	*Siphoviridae*	UC	Cheese	41718	ERZ1464547
vB_EfaS_DELF1	*E. faecalis*	*Siphoviridae*	UC	NI	40248	LC513943
vB_EfaS_IME197	*E. faecalis*	*Siphoviridae*	UC	NI	41098	KT945994
vB_Efm_LG62	*E. faecium*	*Siphoviridae*	UC	Sewage	42236	OP018674
VEsP-1	*Enterococcus* spp.	*Siphoviridae*	UC	River water	39221	MZ333456
